# Scientific publishing in nursing: the role of authors, the journal and the publishing process

**DOI:** 10.1590/1518-8345.0000.4572

**Published:** 2025-07-11

**Authors:** Maria Lucia do Carmo Cruz Robazzi, Regina Aparecida Garcia de Lima, Maria Lúcia Zanetti, Sueli Aparecida Frari Galera

**Affiliations:** 1 Universidade de São Paulo, Escola de Enfermagem de Ribeirão Preto, PAHO/WHO Collaborating Centre for Nursing Research Development, Ribeirão Preto, SP, Brazil.

**Figure:**
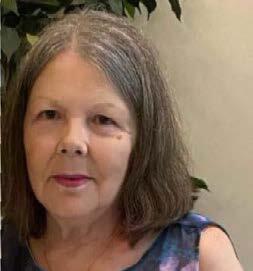

**Figure:**
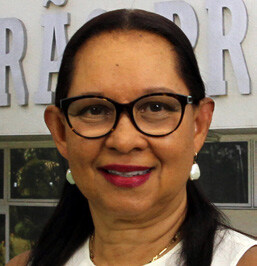

**Figure:**
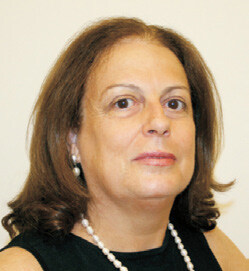

**Figure:**
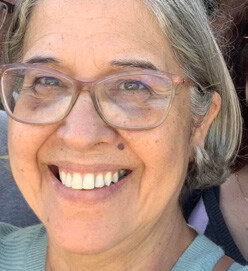

The production of a scientific article is a complex process and depends on several interrelated elements, involving the authors, the content of the text, the journal selected, the publishing process under the responsibility of the journal’s secretariat and editors and the final product, the publication itself. This editorial reports on the experience of some editors with scientific articles in the field of Nursing.

The authors’ decision to prepare a scientific text for submission to a journal starts the process. In order to write this text, in addition to originality in the construction of the object of the study, they seek the use of guidelines, with the aim of increasing the potential for publication, helping to produce more precise texts to support the reproducibility and usefulness of the study carried out^(1)^; in addition, an accurate statistical analysis must be used in quantitative studies^(2)^ and the assumptions anchored in epistemological references for qualitative and mixed studies^(3-4)^.

Authors are also responsible for technical matters, which are no less laborious, such as paying fees and submitting the manuscript with the documents required by the selected journal. After this process, the sometimes long wait for feedback can cause them anguish and stress; for the secretariat team, it results in demands related to monitoring the flow of manuscripts, checking the necessary links if required by the journal, the number of pages, the authors’ affiliation, among other issues, and the text is usually entered into the computerized system adopted by the journal. This stage can be more or less time-consuming, depending on whether all the requirements have been met, since the manuscript may be returned to the authors for adjustments to standards and completeness of documents.

When the manuscript meets the journal’s standards and the documentation is complete, it is sent to the main editors [usually the Editor-in-Chief (ECC) and/or their alternate] who, after analyzing the relevance of the topic; advances in clinical practice, teaching, management and public policy; originality; internal coherence and methodological rigor; main results and conclusions, decide whether or not the manuscript will continue in the evaluation process. This stage also requires more time for analysis, including submitting the text to a similarity checker software in order to identify plagiarism and/or its possible recycling.

Once the previous stages have been completed, the scientific text is sent to one of the journal’s Associate Editors (AE). At this stage, paying attention to open science issues^(5-6)^, the authors are usually already aware of who the AE is assigned to monitor the processing of the article. The AE analyzes the scientific text and forwards it to the reviewers registered in the database of evaluators and, in general, selects three registered reviewers, always obeying the following logic: the evaluator is a specialist in the topic and/or method adopted in the text. In addition to these evaluators, three other alternates are also selected, following the same logic for this selection. Depending on the characteristics of the scientific text, it can also be sent to a statistics professional, usually associated with the journal. The editors’ care with regard to the text submitted also includes checking the registration databases, such as those required for clinical trials, identifying the veracity of the Research Ethics Committee’s declaration, recognizing that good research practices are being followed, among other aspects. This stage requires time and careful review, as a number of problems can compromise the evaluation time.

When the manuscript is returned to the AE, they usually enters their opinion into the system, with or without statistical and/or qualitative evaluation, evaluates the referees’ observations and comments, enters his final evaluation and returns all the texts to the ECC for reconfirmation of his decision. This stage requires time and careful review, as a number of problems can compromise the evaluation time. After this second evaluation by the ECC, the text is sent back to the authors with all the suggestions and/or rejected. If it is sent back, the journal sets the number of days for the return and waits for the authors to reply. When the manuscript is returned with the suggestions accepted and/or justified, it is again re-evaluated by the AE and sent back to the referees for verification of its suitability. At the end, after the evaluators have given their opinion, the text returns to the AE, which recommends (or not) its publication to the ECC.

If approved, the text must be submitted for certification in the language of submission and translated into the other languages, in accordance with the journal’s regulations.

Although the authors, the journal and the publishing process have clear responsibilities, as mentioned above, various factors can compromise this process and extend the deadline. These include authors giving up on resubmitting the manuscript without prior notice, extensive rewriting of the text, delays in resubmission and disagreement with the evaluations of the reviewers, the AE and/or the ECC, which can lead to replications.

Although most journals have databases with the names of reviewers and/or a list of experts - for example through the Web of Science Reviewer Recognition Service - it is often difficult to find one of these experts to do the review. Peer review presupposes credibility, as well as input from the authors; some of the challenges of review relate to the risks of bias, the demand for a long time to evaluate and the quality of the evaluation. To select reviewers, it is important to adopt a strategy of diversifying these experts and to have a database of reliable reviewers^(7)^.

Occasionally, the selected reviewers do not carry out the review, declining this task for numerous reasons. This condition greatly delays the process of getting the scientific text into the journal. It is important to remind authors that their manuscripts need reviewers and that they also need to be available to evaluate other researchers’ manuscripts. Then there are the aspects related to these evaluators who sometimes want to impose their analysis and style on the text being evaluated. Delays in the whole process often occur due to the simultaneous activities of the reviewers; it also sometimes happens that different editors can overload a single reviewer by sending more than one scientific text for their evaluation.

Other general aspects that require attention are sometimes the submission of a scientific text to the journal with grammatical flaws that are at odds with the cultured language required of a scientific article, which is usually pointed out by the editor. Another issue relates to the objective of the study, with discrepancies in the wording of the objective of the abstract compared to that of the body of the text. As for the conclusion, which should refer to the achievement of the objective, it is often the case that it does not allow the reader to know whether the objective has (or has not) been achieved. Another problem observed is that the authors, who are responsible for their texts, often fail to check the final approved versions, which can come back with mistakes. They send the approved article to the translation companies recommended by the journals, but are not always in the habit of checking what has been returned to them. This causes the whole system to slow down, as it waits for the correct final version to be published.

It can be seen that, in the process of publishing articles, failures can occur at all stages, which increases the time between submission and publication of articles, compromising the journal’s immediacy index.

In nursing science, there is an urgent need for more and more valuable, robust articles that are worth reading and have a positive impact on the academic community in general. For this to happen, we also need to have an agile but error-free process, with the active and conscious participation of authors, the technical secretariat, reviewers and editors in this important process of disseminating knowledge.
