# Supplementary material for: Scientific publishing in nursing: the role of authors, the journal and the publishing process
Source: Rev Lat Am Enfermagem. 2025 Jul 11;33:e4572. doi: 10.1590/1518-8345.0000.4572 (PMC12259222; doi:10.1590/1518-8345.0000.4572)
Supplement: Translation

# Translation: Publicação científica em enfermagem: o papel dos autores, do periódico e do processo de editoração

**DOI:** 10.1590/1518-8345.0000.4573

**Authors:** Maria Lucia do Carmo Cruz Robazzi, Regina Aparecida Garcia de Lima, Maria Lúcia Zanetti, Sueli Aparecida Frari Galera

**Affiliations:** 1 Universidade de São Paulo, Escola de Enfermagem de Ribeirão Preto, Centro Colaborador da OPAS/OMS para o Desenvolvimento da Pesquisa em Enfermagem, Ribeirão Preto, SP, Brasil.

A produção de um artigo científico é um processo complexo e depende de vários elementos inter-relacionados, envolvendo os autores, o conteúdo do texto elaborado, o periódico selecionado, o processo de editoração sob a responsabilidade da equipe de secretaria da revista e dos editores e o produto final, a publicação propriamente dita. O presente editorial relata a experiência de alguns editores com os artigos científicos da área de conhecimento de Enfermagem.

A decisão dos autores de elaborar um texto científico para a submissão a um periódico inicia o processo. Para a redação desse texto buscam, além da originalidade na construção do objeto do estudo, a utilização de *guidelines*, com o objetivo de aumentar o potencial de publicação, auxiliando na produção de textos mais precisos para dar suporte à reprodutibilidade e à utilidade do estudo realizado^(1)^; além disso, deve-se ter uma análise estatística acurada nos estudos quantitativos^(2)^ e os pressupostos ancorados em referenciais epistemológicos para estudos qualitativos e mistos^(3-4)^.

Cabe aos autores, também, as providências de ordem técnica, porém não menos trabalhosas, tais como o pagamento de taxas e a submissão do manuscrito com a inclusão dos documentos exigidos pelo periódico selecionado. Após esse processo, a espera pelo retorno dos pareceres, por vezes longa, pode lhes provocar angústia e estresse; para a equipe da secretaria, resulta em demandas relacionadas ao acompanhamento do fluxo dos manuscritos, conferindo os *links* necessários caso seja a exigência da revista, o número de páginas, a afiliação dos autores, entre outras questões e o texto é, em geral, inserido no sistema informatizado adotado pelo periódico. Esta etapa pode ser mais ou menos demorada, dependendo do cumprimento de todas as exigências, uma vez que o manuscrito pode retornar aos autores para ajustes de normas e completude de documentos.

Quando o manuscrito atende às normas do periódico e a documentação está completa, ele é encaminhado aos editores principais [em geral Editor Científico Chefe (ECC) e/ou seu suplente] que, após análise sobre a relevância do tema; avanço na prática clínica, ensino, gestão e políticas públicas; originalidade; coerência interna e rigor metodológico; principais resultados e conclusões, decidem se o manuscrito continuará, ou não, no processo de avaliação. Esta etapa também exige um tempo maior de análise, inclusive para submeter o texto a um *software* revisor de similaridades, buscando identificar plágios e/ou sua possível reciclagem.

Após o cumprimento das etapas anteriores, o texto científico é encaminhado a um dos Editores Associados (EA) do periódico. Nesta etapa, atentando-se às questões da ciência aberta^(5-6)^, em geral os autores já tomam conhecimento de quem é o EA designado para acompanhar a tramitação do artigo. O EA analisa o texto científico e encaminha-o aos revisores cadastrados no banco de dados de avaliadores e, em geral, seleciona três pareceristas cadastrados, obedecendo sempre à seguinte lógica: o avaliador é especialista no tema e/ou no método adotado no texto. Além desses avaliadores, são igualmente selecionados três outros suplentes, obedecendo à mesma lógica para esta seleção. A depender da característica do texto científico, pode-se, também, enviá-lo a um profissional de estatística, geralmente vinculado ao periódico. Os cuidados dos editores em relação ao texto submetido incluem, também, a conferência em bases de registro como, por exemplo, as exigidas aos ensaios clínicos, a identificação da veracidade da declaração do Comitê de Ética em Pesquisa, o reconhecimento do seguimento das boas práticas em pesquisa, entre outros aspectos. Esta etapa requer tempo e cuidadosa revisão, pois uma série de problemas pode comprometer o tempo de avaliação.

Quando o manuscrito retorna ao EA, ele geralmente coloca no sistema o seu parecer, acrescido ou não da avaliação estatística e/ou de caráter qualitativo, avalia as observações e comentários dos pareceristas, coloca a sua avaliação final e devolve todos os textos ao ECC, para a reconfirmação de sua decisão. Após esta segunda avaliação do ECC, o texto é reenviado aos autores com todas as sugestões e/ou rejeitado. Caso seja reenviado, o periódico delimita o número de dias para a devolução e aguarda a resposta dos autores. O retorno do manuscrito com as sugestões acatadas e/ou justificadas é novamente reavaliado pelo EA e reenviado aos pareceristas para a constatação de sua adequação. Ao final, após a manifestação dos avaliadores, o texto retorna ao EA, que recomenda (ou não) ao ECC a sua publicação.

Caso seja aprovado, o texto deve ser submetido à certificação do idioma de submissão e traduzido para os outros idiomas, conforme as normatizações do periódico.

Embora os autores, o periódico e o processo de editoração tenham responsabilidades claras, conforme foi mencionado, vários fatores podem comprometer esse processo e alargar o prazo previsto. Entre eles estão a desistência dos autores de reenviar o manuscrito sem aviso prévio, reformulações extensas do texto, atrasos na ressubmissão e desacordo com as avaliações dos revisores, do EA e/ou do ECC, o que pode levar às réplicas.

Apesar da maioria dos periódicos possuir bancos de dados com os nomes dos revisores e/ou ter como consultar uma lista de *experts* - por exemplo por meio do *Web of Science Reviewer Recognition Service* -, muitas vezes há dificuldades de encontrar-se um desses especialistas para fazer a revisão. A avaliação por pares pressupõe credibilidade, bem como contribuição com os autores; alguns dos desafios da revisão relacionam-se aos riscos de viés, demanda de longo tempo para avaliar e a qualidade da avaliação. Para selecionar revisores, é importante adotar uma estratégia de diversificação desses especialistas e ter um banco de dados de revisores confiáveis^(7)^.

Ocasionalmente, os revisores selecionados não fazem a revisão, declinando desta tarefa por numerosas razões. Tal condição atrasa e muito o processo de tramitação do texto científico no periódico. Cabe lembrar aos autores que seus manuscritos precisam de revisores e eles precisam também ter disponibilidade para avaliação de manuscritos de outros pesquisadores. Ainda, há os aspectos relacionados a estes avaliadores que às vezes querem impor a sua análise e o seu estilo no texto avaliado. Atrasos no processo todo muitas vezes ocorrem em decorrência de atividades simultâneas dos revisores; às vezes acontece, também, que distintos editores podem sobrecarregar um único revisor, enviando mais que um texto científico para sua avaliação.

Outros aspectos gerais que demandam atenção são, por vezes, a submissão do texto científico ao periódico com falhas gramaticais, discordantes da linguagem culta exigida a um artigo científico, o que costuma ser pontuado pelo editor. Outra questão refere-se ao objetivo do estudo, havendo discrepâncias na redação do objetivo do resumo em relação ao do corpo do texto. Quanto à conclusão que deve remeter ao alcance do objetivo, constata-se, muitas vezes, que ela não permite ao leitor conhecer se o objetivo foi (ou não) alcançado. Um outro problema observado é que os autores, responsáveis pelos seus textos, costumam não realizar a conferência das versões finais aprovadas, que podem retornar com equívocos. Eles encaminham o artigo aprovado às empresas de tradução recomendadas pelos periódicos, mas nem sempre têm o hábito de conferir o que lhes foi devolvido. Esse fato favorece uma morosidade em todo o sistema, que fica no aguardo da versão final correta para efetivar a publicação.

Identifica-se que, em processos de editoração de artigos, falhas podem ocorrer em todas etapas, o que amplia o tempo entre a submissão e a publicação dos artigos, comprometendo o índice de imediatez do periódico.

Na ciência da Enfermagem, urge que tenhamos cada vez mais artigos valiosos, robustos, que mereçam ser lidos e tenham repercussões positivas na comunidade acadêmica em geral. Para isso, também, necessitamos ter um processo ágil, mas sem erros, com a ativa e consciente participação dos autores, secretaria técnica, revisores e editores, neste importante processo de divulgação do conhecimento.

# Translation: Publicación científica en enfermería: el papel de los autores, del periódico y del proceso de producción editorial

**DOI:** 10.1590/1518-8345.0000.4571

**Authors:** Maria Lucia do Carmo Cruz Robazzi, Regina Aparecida Garcia de Lima, Maria Lúcia Zanetti, Sueli Aparecida Frari Galera

**Affiliations:** 1 Universidade de São Paulo, Escola de Enfermagem de Ribeirão Preto, Centro Colaborador de la OPS/OMS para el Desarrollo de la Investigación en Enfermería, Ribeirão Preto, SP, Brasil.

La producción de un artículo científico es un proceso complejo y depende de varios elementos interrelacionados, involucrando a los autores, el contenido del texto elaborado, la revista seleccionada, el proceso de edición bajo la responsabilidad del equipo de secretaría de la revista y de los editores, y el producto final, la publicación propiamente dicha. El presente editorial relata la experiencia de algunos editores con los artículos científicos del área de conocimiento de Enfermería.

La decisión de los autores de elaborar un texto científico para la sumisión a un periódico inicia el proceso. Para la redacción de este texto buscan, además de la originalidad en la construcción del objeto del estudio, la utilización de *guidelines*, con el objetivo de aumentar el potencial de publicación, auxiliando en la producción de textos más precisos para dar soporte a la reproductibilidad y a la utilidad del estudio realizado^(1)^; además, se debe tener un análisis estadístico preciso en los estudios cuantitativos^(2)^ y los supuestos anclados en referenciales epistemológicos para estudios cualitativos y mixtos^(3-4)^.

Corresponde a los autores, también, las providencias de orden técnica, pero no menos trabajosa, tales como el pago de tasas y la sumisión del manuscrito con la inclusión de los documentos exigidos por el periódico seleccionado. Tras este proceso, la espera por el retorno de los pareceres, por veces larga, puede provocarles angustia y estrés; para el equipo de la secretaría, resulta en demandas relacionadas al acompañamiento del flujo de los manuscritos, verificando los enlaces necesarios en caso de que sea un requisito de la revista, el número de páginas, la afiliación de los autores, entre otras cuestiones y el texto es, en general, ingresado en el sistema informatizado adoptado por el periódico. Esta etapa puede ser más o menos demorada, dependiendo del cumplimiento de todas las exigencias, una vez que el manuscrito puede retornar a los autores para ajustes de normas y completitud de documentos.

Cuando el manuscrito cumple con las normas del periódico y la documentación está completa, es enviado a los editores principales [en general Editor Científico Jefe (ECC) y/o su suplente] que, tras al análisis sobre la relevancia del tema; avance en la práctica clínica, enseñanza, gestión y políticas públicas; originalidad; coherencia interna y rigor metodológico; principales resultados y conclusiones, deciden si el manuscrito continuará, o no, en el proceso de evaluación. Esta etapa también exige un tiempo mayor de análisis, inclusivo para someter el texto a un *software* revisor de similitudes, buscando identificar plagios y/o su posible reciclaje.

Después del cumplimiento de las etapas anteriores, el texto científico es enviado a uno de los Editores Asociados (EA) del periódico. En esta etapa, atendiendo a las cuestiones de la ciencia abierta^(5-6)^, en general, los autores ya conocen quién es el EA designado para acompañar la tramitación del artículo. El EA analiza el texto científico y lo envía a los revisores registrados en el banco de datos de evaluadores y, en general, selecciona tres revisores registrados, obedeciendo siempre a la siguiente lógica: el evaluador es experto en el tema y/o en el método adoptado en el texto. Además de estos evaluadores, son igualmente seleccionados tres otros suplentes, obedeciendo a la misma lógica para esta selección. A depender de la característica del texto científico, se puede, también, enviarlo a un profesional de estadística, generalmente vinculado al periódico. Los cuidados de los editores en relación al texto sometido prevén, también, la conferencia en bases de registro como, por ejemplo, las exigidas a los ensayos clínicos, la identificación de la veracidad de la declaración del Comité de Ética en Pesquisa, el reconocimiento del seguimiento de las buenas prácticas en pesquisa, entre otros aspectos. Esta etapa requiere tiempo y cuidadosa revisión, pues una serie de problemas puede comprometer el tiempo de evaluación.

Cuando el manuscrito regresa al EA, generalmente ingresa/ en el sistema su parecer, con o sin la evaluación estadística y/o cualitativa, evalúa las observaciones y comentarios de los revisores, coloca su evaluación final y devuelve todos los textos al ECC para la reconfirmación de su decisión. Después de esta segunda evaluación del ECC, el texto es reenviado a los autores con todas las sugerencias y/o rechazado. Caso sea reenviado, el periódico delimita el número de días para la devolución y aguarda la respuesta de los autores. El retorno del manuscrito con las sugerencias acatadas y/o justificadas es nuevamente reevaluado por el EA y reenviado a los revisores para la constatación de su adecuación. Al final, tras la manifestación de los evaluadores, el texto regresa al EA, que recomienda (o no) al ECC su publicación.

Cao sea aprobado, el texto debe ser sometido a la certificación del idioma de sumisión y traducido a los otros idiomas, conforme las normativas del periódico.

Aunque los autores, el periódico y el proceso editorial tengan responsabilidades claras, conforme se ha mencionado, varios factores pueden comprometer este proceso y ampliar el plazo previsto. Entre ellos están la desistencia de los autores de reenviar el manuscrito sin aviso previo, reformulaciones extensas del texto, retrasos en el reenvío y desacuerdo con las evaluaciones de los revisores, del EA y/o del ECC, lo que puede llevar a las réplicas.

A pesar de la mayoría de los periódicos tener bancos de datos con los nombres de los revisores y/o tener la posibilidad de consultar una lista de expertos - por ejemplo por medio del *Web of Science Reviewer Recognition Service* -, muchas veces hay dificultades de encontrar uno de estos expertos para hacer la revisión. La evaluación por pares presupone credibilidad, así como contribución con los autores; algunos de los desafíos de la revisión están relacionados con los riesgos de sesgo, la demanda de tiempo prolongado para evaluar y la calidad de la evaluación. Para seleccionar revisores, es importante adoptar una estrategia de diversificación de estos expertos y tener un banco de datos de revisores confiables^(7)^.

Ocasionalmente, los revisores seleccionados no hacen la revisión, declinando esta tarefa por numerosas razones. Tal condición retrasa y mucho el proceso de tramitación del texto científico en el periódico. Cabe recordar a los autores que sus manuscritos necesitan revisores y ellos también deben tener disponibilidad para evaluar manuscritos de otros investigadores. Además, existen aspectos relacionados con estos evaluadores que a veces quieren imponer su análisis y su estilo en el texto evaluado. Retrasos en el proceso todo muchas veces ocurren en consecuencia de actividades simultáneas de los revisores; a veces acontece, también, que distintos editores pueden sobrecargar un único revisor, enviándole más que un texto científico para su evaluación.

Otros aspectos generales que demandan atención son, por veces, la sumisión del texto científico al periódico con errores gramaticales, discordantes del lenguaje culto exigido a un artículo científico, lo que suele ser apuntado por el editor. Otra cuestión se refiere al objetivo del estudio, si hay discrepancias en la redacción del objetivo del resumen en relación con el del cuerpo del texto. Cuanto a la conclusión que debe remitir al alcance del objetivo, se observa, en muchas ocasiones, que no permite al lector saber si el objetivo fue (o no) alcanzado. Un otro problema observado es que los autores, responsables por sus textos, a menudo no realizan la comprobación/conferencia de las versiones finales aprobadas, que pueden regresar con errores. Ellos envían el artículo aprobado a las empresas de traducción recomendadas por los periódicos, pero ni siempre tienen el hábito de conferir lo que les ha sido devuelto. Este hecho favorece una morosidad en todo el sistema, que queda a la espera de la versión final correcta para efectuar la publicación.

Se identifica que, en procesos editoriales de artículos, errores pueden ocurrir en todas etapas, lo que amplía el tiempo entre la sumisión y la publicación de los artículos, comprometiendo el índice de inmediatez del periódico.

En la ciencia de la Enfermería, urge que tengamos cada vez más artículos valerosos, robustos, que merezcan ser leídos y tengan repercusiones positivas en la comunidad académica en general. Para ello, también, necesitamos tener un proceso ágil, pero sin errores, con la activa y consciente participación de los autores, secretaría técnica, revisores y editores, en este importante proceso de divulgación del conocimiento.
